# Predicting the presence of coronary artery disease by transesophageal echocardiography

**DOI:** 10.1007/s00508-020-01686-x

**Published:** 2020-06-12

**Authors:** Matthias Schneider, Houtan Heidari, Hong Ran, Christian Roth, Christian Hengstenberg, Thomas Binder, Georg Goliasch

**Affiliations:** 1 Department of Internal Medicine II, Medical University of Vienna, Waehringer Guertel 18–20, 1090 Vienna, Austria; 2 Department of Echocardiography, Nanjing First Hospital Affiliated to Nanjing Medical University, Nanjing, China

**Keywords:** TEE, Aorta, Aortic plaque, Mitral annular calcification, Aortic stenosis

## Abstract

**Background:**

The accuracy of ultrasound signs as predictors for the presence of coronary artery disease (CAD) has been evaluated extensively in the 1990s and 2000s. Imaging quality has improved tremendously over the last decades.

**Hypothesis:**

High-end ultrasound systems allow for accurate prediction of the presence or absence of CAD.

**Methods:**

All patients who underwent a transesophageal echocardiography examination (TEE) between 2007 and 2016 and who had coronary angiography within 24 months before or after the TEE were retrospectively evaluated.

**Results:**

A total of 242 patients fulfilled the inclusion criteria, 60% were male. Mean age was 70 years (SD ± 13 years). In multivariate regression analysis, plaque in the ascending aorta (odds ratio [OR] 2.51, 95% confidence interval [CI] 1.18–5.32, *p* = 0.017), plaque in at least one of the thoracic aortic segments (OR 2.07, 95% CI 1.02–4.22, *p* = 0.045), and the presence of mitral annular calcification (MAC, OR 1.84, 95% CI 1.01–3.36, *p* = 0.046) were predictors of significant CAD. The isolated finding of aortic stenosis (AS) (OR 2.53, 95%CI 1.23–5.21, *p* = 0.012) was a significant predictor for the absence of normal coronary arteries.

**Conclusion:**

With an negative predictive value (NPV) of 80%, the absence of MAC, AS, and aortic plaque makes the presence of significant CAD unlikely. If at least mild AS is present, normal coronary arteries are improbable.

## Introduction

The accuracy of ultrasound signs as predictors for the presence of coronary artery disease (CAD) has been evaluated extensively in the 1990s and early 2000s. Imaging quality has changed tremendously since. The data used for earlier analyses were based on ultrasound images, which do not compare to today’s imaging systems, some of the earlier studies on mitral annular calcification (MAC) being based on M‑mode findings [1–3].

Of the patients with peripheral arterial disease 41% have concomitant coronary or cerebrovascular disease [4]. In the Framingham study the presence of MAC predicted all cause death, cardiovascular death, and cardiovascular disease [5]. The presence of MAC was an independent predictor of cardiovascular events in another study, the risk being directly related to severity of MAC [6]. Premature MAC and aortic valve sclerosis (AVS) in young patients were found to be a predictor of reduced myocardial perfusion [7] and for the presence of CAD [8–10]. Exposure to multiple cardiovascular risk factors at a young age is associated with increased prevalence of MAC and AVS later in life [11]. Mitral annular calcification and AS detected in TTE predict the presence of significant CAD [12]. The presence of both MAC and aortic plaques detected in TEE, was associated with a higher incidence of stroke and cardiac death in a patient group investigated in the 1990s [13]. Others could show the association of inflammation and presence of MAC [14] as well as an association between the presence of MAC with AVS, aortic plaque, and CAD [15, 16]; however, in a recent study, the clinical significance of MAC was challenged and it was discussed to be a mere age-related degenerative finding rather than a predictor of cardiovascular disease [17].

The absence of atherosclerosis in the thoracic aorta resulted in a negative predictive value of 90–99% for the presence of significant CAD in a number of previous studies [18–21]. Gu et al. showed that the presence of aortic plaques, especially those in the descending aorta, are associated with the presence of significant CAD [22]. The presence of severe aortic plaque in TEE is a predictor for poor survival as well as a risk for ischemic stroke [23, 24].

The aim of this study was to re-evaluate the predictive accuracy of MAC, AVS, and aortic plaque detected in TEE examination on high-end ultrasound machines for the diagnosis of CAD.

## Methods

### Study population

In this single center retrospective cohort study, all patients who received a TEE examination between 1 January 2007 and 30 June 2016 and who had coronary angiography within 24 months before or after the TEE were evaluated. The ethics committee of the Medical University of Vienna (Nr. 1725/2016) approved the study in accordance with the Declaration of Helsinki.

In the searched period, 242 patients had TEE reports clearly describing the degree of aortic plaque, and the presence or absence of MAC and aortic stenosis (AS), and complete documentation of coronary angiography.

A systematic exploration of the centralized patient management system of Vienna (AKIM-AKH-Informationsmanagement) was performed to obtain information on medical diagnoses and daily medication.

### Echocardiography

Data on echocardiographic examinations were retrospectively extracted from the hospital database. Examinations were performed with the following echo machines: Siemens Acuson (Siemens, Munich, Germany), Philips iE33 (Koninklijke Philips N.V. Amsterdam, Netherlands), Acuson Sequoja 512 (Siemens, Munich, Germany), and GE Vivid 7 (General Electric Healthcare, Chicago, IL, USA). At our institution the morphology of the aorta is visualized and described in every TEE examination.

**Fig. 1:**
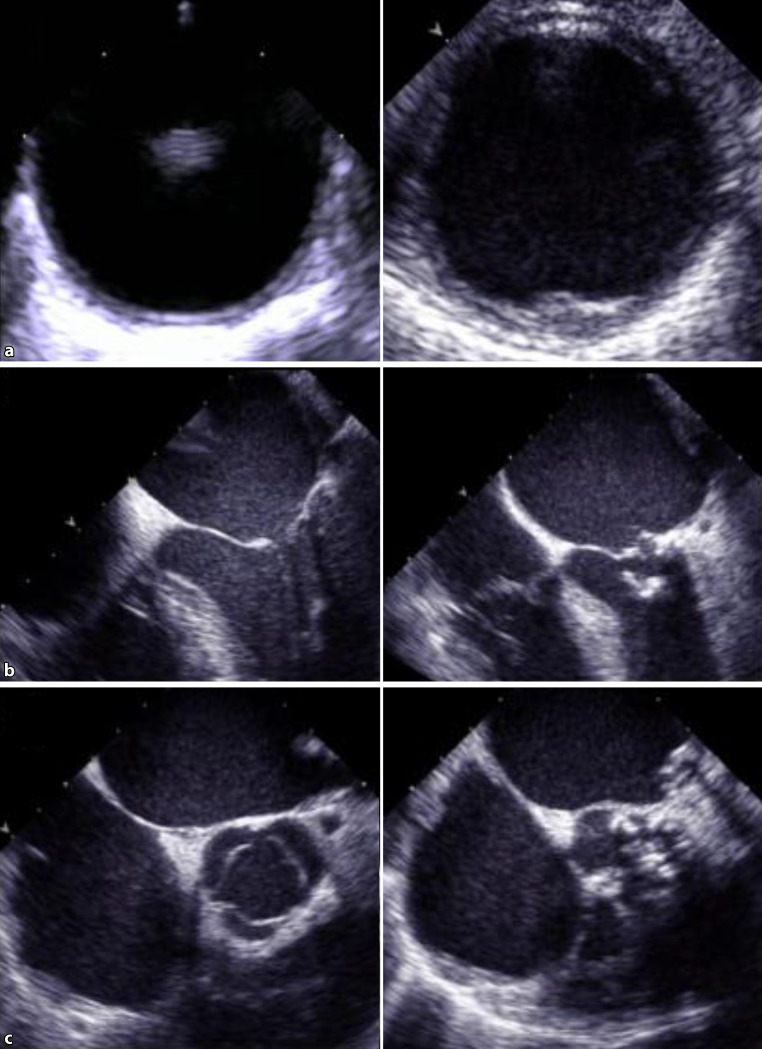
Representative images displaying a transesophageal short axis view of the descending thoracic aorta (panel **a**) without (*left side*) and with (*right side*) aortic plaque, a transesophageal four-chamber view (panel **b**) with normal (*left side*) and severely calcified (*right side*) mitral valve, and a transesophageal short axis view of the aortic valve (panel **c**) with normal (*left side*) and severely calcified (*right side*) morphology

The degree of calcification is reported as no plaque, minimal plaque, minimal to moderate plaque, moderate plaque, moderate to severe plaque, and severe plaque. The differentiation between the groups is made by visual quantification only. To minimize interobserver variability and to reliably identify those patients with relevant aortic plaque, we evaluated two groups, one with minimal or no aortic plaque and one with more than minimal aortic plaque. Presence of MAC was diagnosed by visual assessment and defined as the presence of at least mild calcification of the mitral annulus. We evaluated two groups, one without MAC and one with at least mild MAC. Presence of aortic stenosis (AS) was diagnosed by visual assessment as well as by interpretation of continuous wave Doppler measurements. We evaluated two groups, one without AS and one with at least mild AS (Fig. 1).

### Coronary angiography

We retrospectively extracted reports of the coronary angiograms. Presence of CAD was defined as any lesion in at least one of the coronary arteries. We further divided in the two groups significant and non-significant CAD, while significant obstructive CAD was defined as ≥70% stenosis of ≥1 major branch.

### Statistical analysis

Continuous variables are given as mean ± standard deviation (SD). Categorical data are presented as absolute numbers and percentages. Comparison of categorical data was performed using Student’s unpaired t‑test analysis. A *P*-value of <0.05 was considered statistically significant. Positive and negative predictive values were calculated for the prediction of presence and absence of CAD. Univariate and multivariate logistic regression analyses were applied to examine the relationships between presence and absence of CAD and aortic plaque, MAC, and AS. The multivariate model was adjusted for established cardiovascular risk factors (arterial hypertension, diabetes mellitus, history of smoking, hyperlipidemia, and age) to control for potential confounding. The SPSS Version 24 (IBM SPSS, USA) was used for all analyses.

## Results

### Patient characteristics

At total of 242 patients fulfilled the inclusion criteria, 61% were male. Mean age was 70 (SD ± 13) years. All patients received TEE and coronary angiography within 24 months, mean time between the 2 examinations was 99 days. Most common indications for TEE were preoperative/preinterventional evaluation of valve disease (44.6%), exclusion of intracardiac thrombi (21.9%), and suspicion for endocarditis (14.5%). Indications for coronary angiography were preoperative angiography (59.9%), clinical suspicion of CAD (32.6%) and acute coronary syndrome (6.6%).

Normal coronary arteries were present in 104 (43%) of the patients. Significant CAD was present in 98 (41%) of the patients. Left main stenosis was present in 7 patients, 6 of those had concomitant CAD with at least 1 other stenosis ≥70%, 1 patient had a 90% isolated left main stenosis. In the overall group 119 (49%) patients had plaque in at least 1 part of the thoracic aorta, 87 (36%) had at least mild AS, and 88 (36%) had at least mild MAC. Plaque in the ascending aorta was found in 14%, in the aortic arch in 31%, and in the descending aorta in 28%.

### Patients with coronary artery disease

A total of 138 (57%) patients had CAD. When compared to those without CAD, they were older (73 years vs. 65 years, *p* < 0.001), and arterial hypertension (84% vs. 64%, *p* < 0.001), diabetes mellitus (33% vs. 18%, *p* = 0.012), and hyperlipidemia (59% vs. 23%, *p* < 0.001) were more common. While there was no significant difference in presence of plaque in the aorta, MAC (46% vs. 23%, *p* < 0.001) and at least mild AS (46% vs. 23%, *p* < 0.001) were more frequent (Table 1).

**Table 1:** Patient characteristics, echocardiographic and invasive data (*n* = 242)

	Total	No CAD	CAD	*p*-value
**Patient characteristics**				
Number of patients, *n* (%)	242 (100)	104 (43)	138 (57)	–
Age, mean years (SD)	70 (±13)	65 (±15)	73 (±11)	<0.001
Male sex, *n* (%)	147 (61)	61 (59)	86 (62)	0.565
*Cardiovascular risk factors*				
Arterial hypertension, *n* (%)	183 (76)	67 (64)	116 (84)	<0.001
Diabetes mellitus, *n* (%)	64 (26)	19 (18)	45 (33)	0.012
Hyperlipidemia, *n* (%)	105 (43)	24 (23)	81 (59)	<0.001
Smoking, *n* (%)	72 (30)	25 (24)	47 (34)	0.092
*Medication*				
Anticoagulation, *n* (%)	111 (46)	57 (55)	54 (39)	0.015
Antiplatelet therapy, *n* (%)	124 (51)	30 (29)	94 (68)	<0.001
ACE inhibitor, *n* (%)	173 (72)	63 (61)	110 (80)	0.001
Beta blocker, *n* (%)	163 (67)	63 (61)	100 (73)	0.051
Diuretics, *n* (%)	170 (70)	67 (64)	103 (75)	0.086
**Echocardiographic data**				
Plaque thoracic aorta, *n* (%)	119 (49)	47 (45)	72 (52)	0.058
Plaque AscAo, *n* (%)	33 (14)	9 (9)	24 (17)	0.05
Plaque AoArch, *n* (%)	74 (31)	29 (28)	45 (33)	0.335
Plaque DesAo, *n* (%)	67 (28)	25 (24)	42 (30)	0.056
Aortic stenosis ≥ mild, *n* (%)	87 (36)	24 (23)	63 (46)	<0.001
MAC, *n* (%)	88 (36)	24 (23)	64 (46)	<0.001
**Indication for TOE**				
Evaluation of valve disease, *n* (%)	108 (44.6)	50 (48.1)	67 (48.5)	0.879
Exclusion of intracardiac thrombi, *n* (%)	53 (21.9)	25 (24)	28 (20.3)	0.487
Endocarditis, *n* (%)	35 (14.5)	13 (12.5)	22 (15.9)	0.453
Embolic stroke patient: Search for origin of emboly, *n* (%)	20 (8.3)	5 (4.8)	14 (10.1)	0.128
Suspicion for intracardiac shunt lesion, *n* (%)	13 (5.4)	9 (8.7)	4 (2.9)	0.05
Intracardiac tumor, *n* (%)	4 (1.7)	2 (1.9)	2 (1.4)	0.776
**Indication for CA**				
Preoperative CAD evaluation, *n* (%)	145 (59.9)	69 (66.3)	76 (55.1)	0.071
Clinical suspicion for CAD, *n* (%)	79 (32.6)	32 (30.8)	47 (34.1)	0.599
Acute coronary syndrome, *n* (%)	16 (6.6)	2 (1.9)	14 (10.1)	0.011

*CAD* coronary artery disease, *SD* standard deviation, *AscAo* ascending aorta, *DesAo* descending aorta, *AoArch* aortic arch, *MAC* mitral annular calcification

### Prediction of the presence or absence of significant coronary artery disease

In univariate analysis, plaque in the ascending (odds ratio [OR] 2.23, 95% confidence interval [CI] 1.06–4.7, *p* = 0.034) and descending aorta (OR 2.05, 95% CI 1.03–4.07, *p* = 0.041) as well as in the aortic arch (OR 2.84, 95% CI 1.29–6.28, *p* = 0.01), at least mild AS (OR 1.79, 95% CI 1.05–3.06, *p* = 0.033), at least mild MAC (OR 2.52, 95% CI 1.47–4.33, *p* = 0.001), and any two of the previous in combination (OR 2.3, 95% CI 1.29–4.11, *p* = 0.005) were significant predictors of relevant CAD (at least one 70% stenosis), data not shown.

In multivariate regression analysis, plaque in the ascending aorta (OR 2.51, 95%CI 1.18–5.32, *p* = 0.017), plaque in at least one of the thoracic aortic segments (OR 2.07, 95%CI 1.02–4.22, *p* = 0.045), and the presence of MAC (OR 1.84, 95%CI 1.01–3.36, *p* = 0.046) were predictors of significant CAD. In the presence of MAC, AS, and aortic plaque at the same time, there was no association with the presence of significant CAD (OR 1.15, 95%CI 0.52–2.57, *p* = 0.732). For data on regression analysis regarding the presence of significant CAD *see* Table 2.

**Table 2:** Association of age, sex, cardiovascular risk factors (diabetes, hypertension, hyperlipidemia, smoking), and presence of aortic plaque, mitral annular calcification (MAC), and aortic stenosis (AS) with the presence of significant and with any coronary artery disease (CAD)

Variable	OR	95% CI	*p*-value
Multivariate analysis			
*Presence of significant CAD*			
Plaque in the ascending aorta	2.51	1.18–5.32	**0.017**
Plaque in the aortic arch	2.09	0.81–5.36	0.127
Plaque in the descending aorta	1.6	0.72–3.55	0.244
Plaque in at least one of the aortic segments	2.07	1.02–4.22	**0.045**
At least mild MAC	1.84	1.01–3.36	**0.046**
At least mild AS	1.51	0.79–2.89	0.21
*Presence of any CAD*			
At least mild AS	2.53	1.23–5.21	**0.012**
MAC	1.86	0.97–3.59	0.063
MAC and AS	1.92	0.88–4.19	0.102

*Bold values* indicate statistical significance with a *p*-value <0.05

Negative predictive value (NPV) for the presence of significant CAD was 80% if there was neither MAC, nor AS, nor aortic plaque (Table 3).

**Table 3:** Sensitivity, specificity, negative predictive value (NPV), positive predictive value (PPV), and accuracy of TEE signs of atherosclerosis predicting significant coronary artery disease (CAD)

Prediction of significant CAD by TEE signs of atherosclerosis					
	Sensitivity (%)	Specificity (%)	NPV (%)	PPV (%)	Accuracy (%)
Plaque in the ascending aorta	29 (16–46)	81 (70–90)	66 (61–71)	48 (32–65)	62 (52–71)
Plaque in the aortic arch	74 (60–86)	47 (36–59)	74 (63–83)	47 (41–54)	58 (49–67)
Plaque in the descending aorta	57 (42–71)	60 (50–70)	73 (65–79)	43 (35–52)	59 (51–67)
Plaque in at least one of the aortic segments	72 (61–82)	48 (40–57)	75 (66–82)	45 (40–51)	57 (50–64)
MAC	49 (39–60)	72 (64–79)	68 (63–72)	55 (46–63)	63 (56–69)
AS	44 (34–55)	69 (61–77)	65 (60–69)	49 (41–58)	59 (53–65)
None present	87 (78–93)	34 (26–43)	80 (69–87)	46 (43–50)	55 (48–62)
All present	16 (10–26)	88 (82–93)	62 (59–64)	48 (33–64)	60 (53–66)

*AS* aortic stenosis, *MAC* mitral annular calcification

### Prediction of the presence or absence of normal coronary arteries

In univariate analysis, none of the aortic plaque regions for themselves reliably detected the presence or absence of CAD. In multivariate regression analysis, the isolated finding aortic stenosis (OR 2.53, 95% CI 1.23–5.21, *p* = 0.012) was a significant predictor for the absence of normal coronary arteries, see Table 2.

The PPV for the presence of any CAD was 73% for MAC, 72% for AS, and 76% if both AS and MAC were present, data not shown.

## Discussion

In times of modern imaging systems, it is possible to evaluate the exact morphology of the mitral and aortic valves as well as the wall of the thoracic aorta via TEE in almost every patient. When analyzing these findings of generalized atherosclerosis, it is important to draw the correct conclusions. With our data, we could show that while the absence of MAC, AS, and aortic plaque can predict the absence of significant CAD, and the presence of AS can predict the absence of normal coronary arteries, the presence of severe multifocal generalized atherosclerosis is more a sign of age-related degeneration than a reliable predictor of CAD.

Assessment of plaque in the thoracic aorta as well as of MAC and AS is part of every routine TEE examination [25]. Their presence and their predictive power regarding CAD have been analyzed in numerous studies in the past decades [5–10, 12, 15, 16]. When interpreting previous studies, it is of great importance to differentiate those studies which analyzed TTE images from those which analyzed TEE images. There are two biases that must be considered: one is that determining low-grade MAC or AVS can be challenging by TTE but is easy and reliable in TEE due to superior image quality. The other is that most of the studies cited are retrospective data analyses, which leads to a significant selection bias between one group which received TTE and coronary angiography (bias towards acute coronary syndromes) and the other which received TEE and coronary angiography (bias towards valvular heart disease) in close time intervals. In addition, there has been a shift in technology in recent years. The earlier studies did not only use outdated ultrasound machines for diagnosis but also methods such as M‑mode which do not compare to today’s possibilities with direct visualization of calcifications in TEE imaging.

In the following, two important clinical questions are addressed. One is the diagnostic performance of signs of general atherosclerosis detected in high-end TEE imaging in the exclusion of CAD. The other is the prediction of significant CAD.

### Predicting significant coronary artery disease

The diagnosis of significant CAD has a direct impact on the prognosis of the patient. In our data, plaque in the ascending aorta and plaque detected in at least one segment of the thoracic aorta were strong predictors of significant CAD in multivariate regression analysis. If there were no aortic plaques, NPV for the presence of significant CAD was 75%. MAC and the presence of two out of the three (MAC, AS, aortic plaque) also predicted significant CAD and AS as a single parameter as well as the presence of all three (MAC, AS, aortic plaque) did not predict significant CAD. If there was neither MAC, nor AS, or aortic plaque, NPV for significant CAD was 80%.

Since abnormal flow patterns in congenitally altered aortic valves such as bicuspid aortic valves can cause premature aortic stenosis, there is an overlap between those who develop premature degenerative AS and those who develop AS due to congenitally altered valves. Therefore, AS can be a misleading sign for generalized atherosclerosis. If bicuspid aortic valves can be excluded, AVS was a predictor for CAD in a previous study [8]. This differentiation was impossible in our analysis due to the retrospective nature of the study and may explain why AS was not a significant predictor of the presence of significant CAD in our data.

The presence of MAC, AS, and aortic plaque implies severe generalized atherosclerosis, which can develop due to severe cardiovascular risk profiles or due to advanced age. Interestingly, this pattern is not necessarily associated with the presence of severe CAD. Of the 32 patients with calcifications in all 3 regions, 16 (50%) had significant CAD, and 8 (25%) had normal coronary arteries. Thus, degenerative findings in TEE examination must be interpreted carefully regarding the presence of CAD.

### Excluding coronary artery disease

Negative predictive value of coronary computed tomography angiography (CCTA) for the presence of CAD reaches 97–99% [26–29] but CCTA is less reliable in patients with coronary artery calcification. Diagnostic dilemmas are calcifications detected by CCTA, which later turn out to be non-significant in angiography. In these cases, CCTA exposed the patient to unnecessary contrast agent and radiation. Therefore, the most important differentiation by non-invasive or minimally invasive imaging regarding CAD must be the wise selection of those patients who most likely do not have CAD to steer decision making regarding CCTA versus coronary angiography. In many patients where CAD needs to be excluded, TEE examination has been performed before, e.g. in patients with endocarditis, mitral valve pathologies requiring surgery, and in patients with atrial fibrillation where there is clinical suspicion for CAD.

The current guidelines suggest considering the presence of decreased left ventricular function and regional wall motion abnormalities to decide about further diagnostic tests [30]. Secondary echocardiographic findings such as calcification and sclerosis of valves and vessels are not used for determination of pretest probability for CAD. This is because of conflicting data regarding the association of general degenerative findings with the presence of CAD.

In our data, there was no significant association between the presence of aortic plaque with the presence or absence of CAD. This can be attributed to the fact that our patient collective had a high prevalence of cardiovascular risk factors and in the group with normal coronary arteries, aortic plaque could be found in as many as 28% in the aortic arch and in 24% in the descending aorta; however, the isolated finding of at least mild AS was a significant predictor for CAD in multivariate regression analysis. The PPV for the presence of CAD was 73% for MAC, 72% for AS, and 76% if both AS and MAC were present. If there was MAC, AS, or plaque in at least one segment of the aorta, sensitivity for the presence of any CAD was 83%.

Previous data have shown differing results, partly with significantly better results regarding the prognostic value of echo findings; however, the study designs differed tremendously. Some studies considered a coronary artery stenosis of at least 50% to be significant [18, 19, 21], another study was retrospective and with few patients [20].

In our study, there was an independent association of MAC with the presence of significant CAD in multivariate regression analysis. This is in contrast to previous data, e.g. by Bhatt H et al. [17]. This group retrospectively looked at patients who had received TTE and coronary angiography within 3 years. It can be assumed that this study included a different patient population than ours, e.g. patients with acute coronary syndrome. In our cohort, only 6.6% of patients presented with ACS. The majority were evaluated for elective valvular heart surgery/intervention. It can be concluded that prospective data are needed to clarify the relevance of MAC and other signs of atherosclerosis in the prediction of CAD.

These data can be understood as hypothesis generating: If there are no signs of atherosclerosis in the TEE examination performed for the preinterventional or preoperative evaluation of valvular heart disease, CCTA might be sufficient for exclusion of CAD independent of the age of the patient.

### Limitations

This study has limitations. The data collection was retrospective; the reported values were exported from the hospital database. A cohort which has received both coronary angiography and TEE examination at a tertiary medical center within 24 months cannot be compared to the normal population; however, despite the high prevalence of generalized atherosclerosis in our patient group, there were nevertheless significant differences between the analyzed groups. A prospective study with careful inclusion of participants is needed to clarify this bias, which has certainly affected the previously published retrospective studies as well.

## Conclusion

With modern ultrasound systems, the presence of MAC, AS, and aortic plaque can be examined reliably in almost all TEE examinations. With an NPV of 80%, their absence makes the presence of significant CAD unlikely. If at least mild AS is present, normal coronary arteries are improbable.
